# Exocytosis of serotonin from the neuronal soma is sustained by a serotonin and calcium-dependent feedback loop

**DOI:** 10.3389/fncel.2014.00169

**Published:** 2014-06-27

**Authors:** Carolina Leon-Pinzon, Montserrat G. Cercós, Paula Noguez, Citlali Trueta, Francisco F. De-Miguel

**Affiliations:** ^1^Instituto de Fisiología Celular-Neurociencias, Universidad Nacional Autónoma de MéxicoMéxico D.F., México; ^2^Departamento de Neurofisiología, Instituto Nacional de Psiquiatriìa Ramoìn de la Fuente MunñizMéxico D.F., México

**Keywords:** exocytosis, extrasynaptic, somatic exocytosis, extrasynaptic release, serotonin, 5-HT, calcium, positive feedback

## Abstract

The soma of many neurons releases large amounts of transmitter molecules through an exocytosis process that continues for hundreds of seconds after the end of the triggering stimulus. Transmitters released in this way modulate the activity of neurons, glia and blood vessels over vast volumes of the nervous system. Here we studied how somatic exocytosis is maintained for such long periods in the absence of electrical stimulation and transmembrane Ca^2+^ entry. Somatic exocytosis of serotonin from dense core vesicles could be triggered by a train of 10 action potentials at 20 Hz in Retzius neurons of the leech. However, the same number of action potentials produced at 1 Hz failed to evoke any exocytosis. The 20-Hz train evoked exocytosis through a sequence of intracellular Ca^2+^ transients, with each transient having a different origin, timing and intracellular distribution. Upon electrical stimulation, transmembrane Ca^2+^ entry through L-type channels activated Ca^2+^-induced Ca^2+^ release. A resulting fast Ca^2+^ transient evoked an early exocytosis of serotonin from sparse vesicles resting close to the plasma membrane. This Ca^2+^ transient also triggered the transport of distant clusters of vesicles toward the plasma membrane. Upon exocytosis, the released serotonin activated autoreceptors coupled to phospholipase C, which in turn produced an intracellular Ca^2+^ increase in the submembrane shell. This localized Ca^2+^ increase evoked new exocytosis as the vesicles in the clusters arrived gradually at the plasma membrane. In this way, the extracellular serotonin elevated the intracellular Ca^2+^ and this Ca^2+^ evoked more exocytosis. The resulting positive feedback loop maintained exocytosis for the following hundreds of seconds until the last vesicles in the clusters fused. Since somatic exocytosis displays similar kinetics in neurons releasing different types of transmitters, the data presented here contributes to understand the cellular basis of paracrine neurotransmission.

## Introduction

In addition to the canonical release of transmitters from synapses, many neuron types release transmitters or peptides by exocytosis from their soma (For review see Trueta and De-Miguel, 2012). A brief train of impulses at high frequency or a long depolarization evoke a “large-scale” somatic exocytosis, in which large amounts of transmitter molecules are released for hundreds of seconds (Puopolo et al., 2001; Trueta et al., 2003; Soldo et al., 2004; Zhang et al., 2007; Kaushalya et al., 2008). This release seems to be responsible for the modulation of many functions in the nervous system (for review see Trueta and De-Miguel, 2012). Since somatic exocytosis depends on transmembrane Ca^2+^ entry (Sun and Poo, 1987; Chen et al., 1996; Jaffe et al., 1998; Puopolo et al., 2001; Trueta et al., 2003; Soldo et al., 2004; Huang et al., 2007; Kaushalya et al., 2008; Hirasawa et al., 2009), one wonders how it is maintained for such long periods after electrical stimulation and transmembrane Ca^2+^ entry have ended.

To study this problem we took advantage of the serotonergic Retzius neurons of the leech, in which most fine mechanisms of serotonin (5-HT) exocytosis from the synapses and soma were first elucidated (For review see Nicholls and Kuffler, 1990; De-Miguel and Trueta, 2005). The size of these neurons and the possibility to isolate them and keep them in culture provide excellent experimental conditions to understand cellular principles of 5-HT neurotransmission that have later been confirmed in other neuron types, including those of mammals (for review see Trueta and De-Miguel, 2012). The large soma (60–80 μm diameter) of Retzius neurons contains 5-HT packaged in 100-nm diameter dense core vesicles (Coggeshall, 1972; Bruns et al., 2000). At rest, hundreds of these vesicles are tightly assembled in clusters that remain at a distance from the plasma membrane (De-Miguel et al., 2012; Trueta and De-Miguel, 2012). In response to a train of 10 action potentials at 20 Hz, a microtubule-based transport mobilizes 80–110 of these vesicle clusters to different spots of the plasma membrane (Trueta et al., 2004, 2012; De-Miguel et al., 2012) where all the vesicles in each cluster undergo exocytosis within the following 100–400 s (De-Miguel et al., 2012). Other vesicle clusters may fuse later at the same plasma membrane spot in response to the same stimulation train (De-Miguel et al., 2012). We have estimated that a single 20-Hz train evokes exocytosis from 60,000 to 100,000 vesicles/soma. By contrast, stimulation with 10 impulses delivered at 1 Hz fails to evoke this large-scale exocytosis, and in electron micrographs the vesicle clusters appear at their resting positions (Trueta et al., 2003; De-Miguel et al., 2012). Somatic exocytosis is abolished by blocking transmembrane Ca^2+^ entry through L-type Ca^2+^ channels (Trueta et al., 2003), or reduced by blocking Ca^2+^-induced Ca^2+^ release (Trueta et al., 2004). However, how Ca^2+^ entry evokes such delayed somatic exocytosis and how exocytosis is sustained yet need to be explored. The answer to these questions would provide the cellular basis to understand the wider phenomenon of the paracrine modulation of the nervous system.

In this study we dissected the steps from electrical stimulation until the end of the large-scale somatic exocytosis. Exocytosis was stimulated by a train of 10 action potentials at 20 Hz upon intracellular injection of current pulses. The kinetics of exocytosis from single vesicle clusters was accurately measured from the cumulative fluorescence of FM styryl dyes as vesicles fused with the plasma membrane and were retrieved just below the neuronal surface (Betz and Bewick, 1992). This was possible, since upon endocytosis the vesicle clusters remain in the submembrane shell for minutes before being recycled (Trueta et al., 2012). The amount of exocytosis was quantified as the number of FM1-43 fluorescent spots/soma (Betz and Bewick, 1992), which indicates the number of vesicle clusters that underwent exocytosis. The kinetics of the intracellular Ca^2+^ changes was measured from fluorescence increases of Ca^2+^-sensitive dyes injected intracellularly (Gee et al., 2000).

## Materials and methods

### Ethics statement

Animal research was conducted according to the statements of the Animal Committee of the Instituto de Fisiología Celular, UNAM, México.

### Isolation and culture of neurons

Imaging experiments were performed in cultured Retzius neurons of leeches *Hirudo verbana* (Siddall et al., 2007). Individual neurons were isolated by suction through a glass pipette (Dietzel et al., 1986). Neurons were then rinsed several times in L-15 culture medium (Sigma-Aldrich) supplemented with 6 mg ml^−1^ glucose, 0.1 mg ml^−1^ gentamicin and 2% heat- inactivated fetal bovine serum and then plated on glass-bottomed culture dishes pre-coated with concanavalin-A (Sigma-Aldrich). Experiments were performed at 18°C after 1–8 days in culture.

### Electrical stimulation and recording

Electrical stimulation consisted of trains of 10 action potentials produced by 10-ms current pulses delivered at 1 or 20 Hz (Trueta et al., 2003; De-Miguel et al., 2012) through a borosilicate microelectrode with a resistance of 18–30 MΩ when filled with 2 M potassium acetate (KAc). The amplitude of the current pulses was adjusted in every neuron between 5 and 8 nA, so that each pulse would produce one action potential. The neuronal resting potential was maintained at −60 mV by direct current injection. Electrical recordings were acquired by an intracellular amplifier AxoClamp 2B (Axon Instruments) connected to an analog-to-digital board Digidata 1200 (Axon Instruments) that acquired at a sampling frequency of 20 KHz using pCLAMP9 software (Axon Instruments). Data were stored in a PC.

To test for possible 5-HT activation of transmembrane currents, voltage clamp and transmembrane current recordings were made under discontinuous single-electrode voltage-clamp conditions. The quality of the clamp conditions was tested by continuously monitoring the time constant of the system at fast speed. The voltage was clamped at −60 mV and stimulation consisted of 10 voltage pulses of 10 ms to +10 mV at 20 Hz. Action potentials during the 20-Hz train could not be clamped. However, good voltage clamp conditions were obtained after the train and there were no significant current changes in the minutes following stimulation.

### Measurements of exocytosis

We measured the cumulative fluorescence increase of FM styryl dyes (Molecular Probes) produced by the progressive exo/endocytosis of dense core vesicles in the clusters (Betz and Bewick, 1992; De-Miguel et al., 2012). FM1-43 (Molecular Probes) was used for measuring only exocytosis and FM4-64 (Molecular Probes) was used in combination with Fluo-4 for simultaneous measurements of exocytosis and Ca^2+^. Both compounds were added to the bath at a final 2 μM dilution and 10 min later neurons were impaled and hyperpolarized to −60 mV by DC current injection to avoid spontaneous firing. The values of exocytosis were measured as the number of FM1-43 spots/soma normalized to the 91 spots/soma produced in response to 20-Hz stimulation. These data were obtained from previous experiments (Trueta et al., 2003, 2004). The reason for using our previous data was that due to their photo-damage equivalent spot-counting could not be made in the neurons that had been used for kinetic measurements. The counts in response to 20-Hz stimulation were considered as 100%, since at this frequency somatic exocytosis reaches its saturation levels (Trueta and De-Miguel, in preparation).

### Measurements of intracellular Ca^2+^ increases

Relative changes in the intracellular Ca^2+^ concentration were measured with the Ca^2+^-sensitive fluorescent dyes Fluo-4 and Fluo-5F pentapotassium salt (Molecular probes). An advantage of these Fluo dyes is that both can be excited with a single wavelength and therefore allow a fast (100 ms/image) imaging rate and /or long acquisition periods when compared with ratiometric dyes, thus reducing bleaching and neuron damage. This was particularly critical for our experiments when imaging simultaneously Ca^2+^ and exocytosis, since two images were acquired for each time point over several minutes. To calibrate the Ca^2+^ concentrations we also tried Fura-2 imaging. However, the high sampling speed necessary to detect the fast Ca^2+^ transient required to double the imaging rates and neurons in these conditions were damaged rapidly by the Fura-2 excitation and emission wavelengths. For this reason we sacrificed the concentration measurements to correlate the precise kinetics of the Ca^2+^ transients with those of exocytosis. Dyes had to be injected by iontophoresis since Retzius neurons do not retain esterase-coupled dyes. For this, the dyes were dissolved in water at a concentration of 5 mM and were injected into the soma by a 4 nA hyperpolarizing current for 4-6 min using borosilicate microelectrodes. The electrode tip was back-filled with the dye solution and the microelectrode was then filled with 0.1 M KCl. Imaging was made 10–15 min after the dye had been loaded.

### Pitfalls and considerations in the use of Ca^2+^ sensitive dyes

Although we detected Ca^2+^ spikes in response to individual action potentials with both Fluo dyes, the low affinity (2.5 μM) Fluo-5F could not detect the small Ca^2+^ transient described in the results sections. For this reason most of the experiments reported here were made by using the higher affinity (Kd = 345 nM) Fluo-4, with which we consistently detected the small Ca^2+^ transient. A major concern of the use of high affinity dyes for our purposes is the possibility that dye saturation distorts the kinetics of the Ca^2+^ dynamics and exocytosis. However, that the kinetics of the fast Ca^2+^ transient obtained with either Fluo dye were identical suggested that Fluo-4 was not saturated under our experimental conditions. This was further tested by the addition of ionomycin (5 μM) to neurons injected with high affinity dye after recording the transient evoked by 20 Hz stimulation. Ionomycin produced fluorescence increases to levels high above the peak fluorescence of the Ca^2+^ transients. In addition, neurons that became damaged during the experiments had similar fluorescence increases. These evidences indicate that the somatic Ca^2+^ increases in response to electrical stimulation were not produced under dye-saturation conditions.

We also assume that any distortion of the intracellular Ca^2+^ kinetics due to buffering by Fluo-4 did not affect significantly our results, since such distortions occur within miliseconds (Sala and Hernández-Cruz, 1990; Neher and Augustine, 1992; Sabatini and Regehr, 1998) while the time courses of the Ca^2+^ transients we recorded ranged from hundreds of miliseconds to minutes. Moreover, the time courses of these distortions are usually faster than our imaging sampling speed. For all these reasons, the Ca^2+^ kinetics we report seem accurate descriptions of the intracellular Ca^2+^ dynamics. It is relevant also to mention here that the kinetics of exocytosis were similar with and without injection of Ca^2+^ sensitive dye. This indicates that Fluo-4 is not producing significant changes in the Ca^2+^ or exocytosis dynamics. In this regard, somatic exocytosis in our experiments responded in the presence of high affinity dyes similarly to chromaffin cells loaded with Fura-2 (Augustine and Neher, 1992; Neher and Augustine, 1992; Chow et al., 1996; Klingauf and Neher, 1997), in which exocytosis also occurs from dense core vesicles and is slower than in synapses.

### Fluorescence imaging

For experiments using a single dye individual neurons were viewed at their soma equator with a Nikon Eclipse TE 200 inverted microscope through a Nikon 100X oil-immersion objective (NA 1.40). Fluorescence measurements of FM1-43 or Fluo-4 were performed with excitation and emission wavelengths at 488 and 535 nm respectively. A cooled CCD camera (IMAGO, Till Vision) acquired image sequences of 640 × 480 pixels. To study fast Fluo-4 fluorescence transients in response to the stimulation trains, images were acquired every 100 ms for 60 s. To follow FM styryl dye changes or the long-lasting Fluo-4 transients, separate images were acquired every 2 s for 15 min. The image sequences were stored digitally by using TILLvisION software.

For simultaneous recordings of exocytosis and Ca^2+^ signals, confocal imaging of FM4-64 and Fluo-4 fluorescence was carried out using an Olympus Fluoview FV1000 upright confocal scanning microscope using 473 nm for Fluo-4 excitation and 560 nm for FM4-64 excitation. For the simultaneous imaging fluorescence was acquired with a 60X water immersion objective (1.1 NA). Fluorescence was detected in parallel by a spectral-based detector capturing 503–543 nm for the Fluo-4 emission and 600–700 nm for the FM4-64 emission. Time-lapse sequences were made by acquiring an image every 2 s for 20 min. Images were stored digitally by using Fluoview 3.1 software (Olympus).

### Image analysis

We used Image-J software (National Institutes of Health) for most of the analysis. The time series of images were aligned with the StackReg plug-in. The mean fluorescence intensity was measured along time or space sequences from 1.8 to 2 μm^2^ regions of interest (ROIs) that contained a fluorescent spot. These areas were chosen because they correspond to the average area of a vesicle cluster (Trueta et al., 2003; De-Miguel et al., 2012). To calculate the fluorescence changes relative to the resting fluorescence, the average intensity in the 20 frames before stimulation (F_0_) was subtracted from the intensity of that ROI at each time [F(t)]. The difference was divided by F_0_, to generate ΔF/F_0_. For simplicity, throughout the text and figures this normalization is referred to as dF/F. Surface plots were made by using an RGB 8 bit color scale calibrated in the dynamic interval. Plotting and curve fitting were performed using Igor Pro 6.2 software (Wavemetrics). Data are presented as mean ± s.e.m. Statistical comparisons were performed using unpaired two tailed Student's *t*-test.

### Pharmacology

To block L-type Ca^2+^ channels we used nimodipine (10 μM; Sigma-Aldrich); to eliminate Ca^2+^-induced Ca^2+^ release we used a combination of ryanodine (100 μM, Invitrogen) and thapsigargin (1.6 μM; Invitrogen). 5-HT receptors were blocked with methysergide (140 μM; Sigma-Aldrich) and to block PLC neurons were preincubated for 30 min with U-73122 (10 μM; Sigma-Aldrich). All drugs were applied to the bathing fluid from 500X stock solutions 15 min before images were taken, except for U-73122, which was added 30 min before.

### Electron microscopy

The general procedures were as in Trueta et al. (2012). In brief, neurons in isolated segmental ganglia were stimulated with 10 trains of 10 impulses at a frequency of 1 Hz delivered at 1 min intervals. Ganglia were then perfused with 0.08 M cacodylate buffer (pH 7.4; Sigma-Aldrich) and fixed for 60 min with 0.6% glutaraldehyde and 0.4% paraformaldehyde, followed by post-fixation for 60 min in 1.0% osmium tetroxide (Kuffler et al., 1987). Thin (70–100 nm) sections were observed in a JEOL 1010 electron microscope (JEOL USA Inc.). Electron micrographs were digitized at 1200 dpi in CMYK mode. For illustration purposes endoplasmic reticulum (ER) was pseudo-colored by using the fast selection and the color equilibrium tools of Photoshop (Adobe).

## Results

### Kinetics of somatic exocytosis and intracellular Ca^2+^

**Figure 1:**
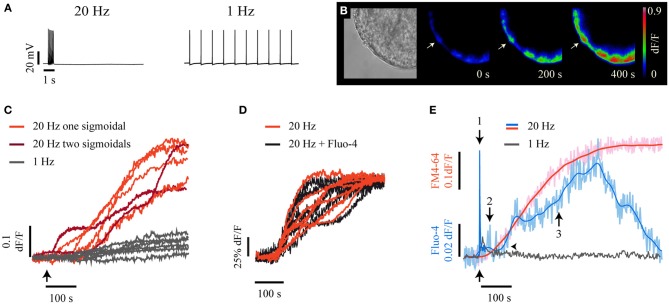
**High frequency electrical stimulation evokes long-lasting somatic exocytosis and Ca^2+^ signals. (A)** Intracellular recordings of trains of 10 impulses at 20 Hz (left) and 1 Hz (right) produced upon intracellular injection of current pulses. **(B)** Time course of FM1-43 fluorescence increases. On the left is a bright field equatorial image of a soma. Scale bar = 10 μm. The sequence on the right shows FM1-43 fluorescence increases at 200 and 400 s following 20-Hz electrical stimulation (time = 0 s). The arrow points to a well-delimited fluorescent spot of the sort used for our kinetic measurements. The color scale on the right indicates the fluorescence levels in arbitrary units. **(C)** Kinetics of FM1-43 fluorescence from six individual spots, each produced by a different neuron in response to 20-Hz stimulation. The arrow indicates the onset of stimulation. The light-red kinetics were described by one sigmoidal, while the two dark-red kinetics were described by two or three sigmoidal steps. The gray traces were obtained from equivalent membrane areas from six other neurons stimulated at 1 Hz. These neurons did not form fluorescent spots in response to electrical stimulation. **(D)** The kinetics of FM4-64 fluorescence upon 20 Hz stimulation were similar in neurons loaded with Fluo-4 (black traces) or in the absence of the Ca^2+^-sensitive dye (red traces). **(E)** Kinetics of simultaneously acquired FM4-64 (pink) and Fluo-4 (pale blue) confocal fluorescence following 20-Hz stimulation. The smoothed kinetics were superimposed in each case (red and blue traces). The Ca^2+^ transient started as a fast transient (1) immediately after the onset of stimulation (black arrow below the traces). The fast Ca^2+^ transient was followed first by a small Ca^2+^ transient (2) and afterwards by a large Ca^2+^ transient (3) that developed during exocytosis. The horizontal arrowhead shows that the large Ca^2+^ transient started after the onset of the FM4-64 increase that indicated the large-scale exocytosis. The large Ca^2+^ transient had its peak a few seconds before the end of exocytosis, seen as the plateau of the FM4-64 fluorescence kinetics. The gray trace is the Fluo-4 fluorescence in response to a 1-Hz train.

Electrical stimulation of individual neurons with a 20-Hz train (Figure 1A, left) lasting 0.5 s produced somatic exocytosis for up to 400 s, as seen by the kinetics of the formation of fluorescent FM1-43 spots in the soma periphery (Figure 1B). Each spot was formed by the integrated fluorescence upon exocytosis from the vesicles in one cluster (De-Miguel et al., 2012). The fluorescence kinetics of 61% of the spots studied in 19 neurons had a sigmoidal shape (Figure 1C, light red traces). The rest of the spots displayed two and occasionally three sigmoidal fluorescence increases (Figure 1C, dark red), each of which was due to the fusion of a subsequent vesicle cluster. We have previously shown (De-Miguel et al., 2012) that the sigmoidal shape of the fluorescence kinetics has the following components: its latency is determined by the resting distance from the vesicle clusters to the plasma membrane and by the transport velocity of the vesicle cluster; the slope of the fluorescence increase expresses the rate of exocytosis; the plateau is reached upon the end of exocytosis, and the maximum fluorescence level is proportional to the cumulative number of vesicles that fused. As expected from our previous experience, stimulation with a 1-Hz train failed to produce a significant formation of FM1-43 fluorescence spots (Figure 1C, gray traces). Consistently, electron micrographs of neurons stimulated at this frequency showed the vesicle clusters at their resting positions (Supplementary Figure 1).

To explore how Ca^2+^ contributes to this large-scale somatic exocytosis, the kinetics of exocytosis and intracellular Ca^2+^ were measured simultaneously by imaging the fluorescence of FM4-64 and Fluo-4 dyes. A major concern of these measurements is that high affinity Ca^2+^ sensitive dyes may affect the intracellular free Ca^2+^ concentration and therefore exocytosis. However, as shown in Figure 1D, the kinetics of exocytosis in neurons injected with Fluo-4 was similar to that obtained from neurons imaged in its absence. The methods section contains an account of the precautions taken for the use of Ca^2+^ sensitive dyes to measure Ca^2+^ dynamics and exocytosis simultaneously. Moreover, as will be seen below, there was also good correlation between the kinetics of exocytosis and intracellular Ca^2+^ in pharmacological experiments.

Simultaneous confocal imaging of FM4-64 and Fluo-4 fluorescence from membrane areas that exhibited exocytosis in response to a 20-Hz train showed a complex relationship between exocytosis and Ca^2+^ (Figure 1E). A 20-Hz train produced a sequence of three Ca^2+^ transients, each of which could be unmistakably identified by its kinetics, its characteristic localization and its effect on exocytosis. The first Ca^2+^ transient was a fast spike coupled to the stimulation train (thus we will refer to it as fast Ca^2+^ transient; 1 in Figure 1E). This fast Ca^2+^ transient invaded rapidly the whole somatic cytoplasm (see Movie 1). The second Ca^2+^ transient was small and purely submembrane (we will refer to it as small Ca^2+^ transient). This small Ca^2+^ transient appeared between the peak of the large Ca^2+^ transient and the large-scale exocytosis (2 in Figure 1E). The third Ca^2+^ transient was large in amplitude and duration (therefore, we will refer to it as large Ca^2+^ transient). The large transient was also restricted to the submembrane shell (3 in Figure 1E), grew during the dynamic range of exocytosis and started to decay when exocytosis was near end. The small and the large Ca^2+^ transients were absent upon 1-Hz stimulation (Figure 1E, gray trace).

### Frequency-dependence of the fast Ca^2+^ transient

**Figure 2:**
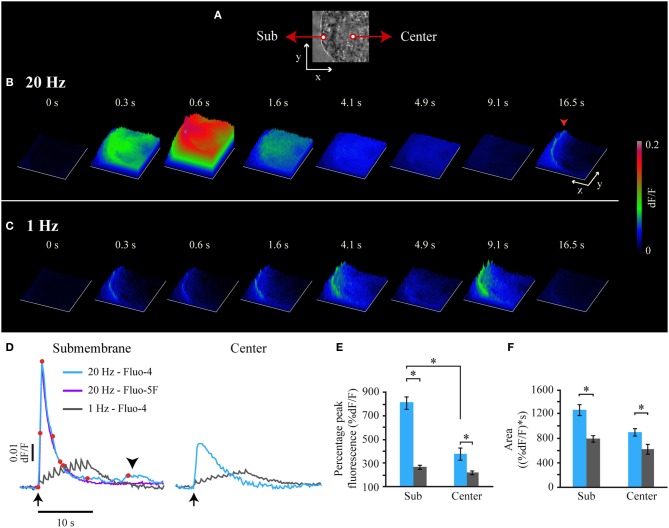
**Frequency-dependence of the fast Ca^2+^ transient. (A)** Bright field image of the soma from which the fluorescence surface plots in **(B,C)** were made. The x and y axis = 20 μm. **(B)** Sequence of Fluo-4 fluorescence surface plots obtained after 20-Hz stimulation at *t* = 0 s. The time is the same for **(B,C)** and corresponds to the time indicated by the red dots over the fast Ca^2+^transient upon 20 Hz stimulation shown in (D). The z axis is the fluorescence intensity in arbitrary units, according to the calibration on the right. At 0.6 s the fast Ca^2+^ transient had reached the soma center. A submembrane small Ca^2+^ transient appeared 16.5 s after the stimulation train (red arrowhead). See also the supplemental Movie 1. **(C)** Sequence of Fluo-4 fluorescence surface plots upon 1-Hz stimulation. This Ca^2+^ increases failed to invade the soma center. See also the supplemental Movie 2. **(D)** Superimposition of Fluo-4 and Fluo-5F fluorescence transients upon 20-Hz stimulation (blue and purple respectively). The arrow pointing up indicates the onset of stimulation. The fluorescence kinetics with both dyes were identical, indicating that Fluo-4 was not saturated. Note also that the small Ca^2+^ transients (arrowhead) were not detected by the low affinity Fluo-5F. A transient in response to 1-Hz (gray) stimulation in the submembrane (left) and central (right) regions was composed by 10 subsequent spikes, each synchronized to an action potential of the train. The transients produced by either stimulation frequency arrived attenuated at the soma center. **(E)** Normalized peak amplitude of the Ca^2+^ transients in response to 20-Hz (blue) and 1-Hz (gray) trains in the submembrane (Sub) and center areas of the soma (Center). The asterisks indicate significant differences (*p* < 0.05). **(F)** Integral values of the Ca^2+^ signals at either frequency in the submembrane and center areas. The larger integral of the transient upon 20-Hz stimulation indicates a supra-linear intracellular Ca^2+^ increase.

Although the fast Ca^2+^ transient ended before the onset of the large-scale exocytosis, the evidence presented before suggested that it coupled electrical stimulation with exocytosis. To understand this paradox we compared its characteristics with to those of the Ca^2+^ transient in response to a 1-Hz train, which does not evoke exocytosis (Figure 2). The fast Ca^2+^ transient upon 20-Hz stimulation started to develop right after the first impulse and peaked 600 ms later (Figures 2B,D, Movie 1), that is ~100 ms after the last impulse (note that these values are approximations due to our 100-ms imaging interval). The peak amplitude of the fast Ca^2+^ transient recorded by Fluo-4 in the submembrane shell was 804 ± 52% of the baseline fluorescence value and its decay was exponential (*R*^2^ > 0.90) with a time constant of 2.0 ± 0.2 s (*n* = 6 cells; Figure 2D). This kinetics was identical when recorded with Fluo-5F or Fluo-4, thus indicating that Fluo-4 did not saturate in these experimental conditions (Figure 2D). However, a major difference was that the fluorescence levels detected by Fluo-5F always returned to baseline levels without unveiling the small Ca^2+^ transient detected by Fluo-4 during the decay of the fast Ca^2+^ transient (Figure 2D, arrowhead). The fast Ca^2+^ transient arrived rapidly at the soma central region (25 μm away from the plasma membrane) in every neuron tested, where it also peaked by 600 ms (Figures 2B,D). However, at the center its peak amplitude was 54% of that in the submembrane shell. From then fluorescence returned exponentially to baseline levels (Figure 2D).

The Ca^2+^ transient produced by a 1-Hz train was different (Figures 2C,D; see Movie 2). In the submembrane shell of the soma this transient was a succession of 10 small Ca^2+^ spikes, with each coupled to an impulse of the train. The peaks of the individual spikes had a ~100 ms rise time (*n* = 6), again with the resolution limited by our 100 ms/image sampling rate. The temporal summation of the subsequent Ca^2+^ spikes produced that the maximal Fluo-4 fluorescence value (263 ± 15% of the baseline value) was reached after the last impulse of the train. The maximum fluorescence amplitude was only 33% of that of the fast Ca^2+^ transient upon 20-Hz stimulation. After the last spike of the 1 Hz train, fluorescence decayed exponentially with a time constant of 1.7 ± 0.3 s. This decay time was similar (p = 0.32) to that of the fast Ca^2+^ transient upon 20-Hz stimulation. At the soma center the maximal amplitude of the transient evoked by 1-Hz stimulation was 72% of that in the periphery.

That the amplitude of the Ca^2+^ transient evoked by 20-Hz stimulation was 67% larger than that obtained at 1-Hz (Figure 2E) suggested that it is the peak amplitude of the Ca^2+^ transient (which indicates the maximum Ca^2+^ concentration reached) what triggers exocytosis. Moreover, the amplitude of the transient evoked by 20-Hz stimulation was 38.5% larger than the sum of the peak amplitudes of the 10 Ca^2+^ spikes evoked by a 1-Hz train. Since transmembrane Ca^2+^ entry upon subsequent voltage steps does not facilitate in the soma of these neurons (Stewart et al., 1989), the supra-linear amplitude increase of the transient evoked at 20-Hz suggests that extracellular Ca^2+^ entry evokes intracellular Ca^2+^ release. Note that the transients produced by either stimulation frequency had the same decay time, thus indicating that the Ca^2+^ buffering in response to both stimulation frequencies was similar and operated within its dynamic range. In these conditions, the integral values of the transients over time provide a measure of the amount of the free Ca^2+^ during the transients regardless on the Ca^2+^ origin and kinetics. As seen in Figure 2F, the integral value of the transients evoked by 20-Hz stimulation was 27% larger than that evoked by 1-Hz stimulation. That the amplitude differences were much larger than the integral differences supports that it is the maximum Ca^2+^ concentration reached and not the amount of free Ca^2+^ what activates exocytosis. This data also supports the idea that Ca^2+^ entry upon 20 Hz stimulation evokes Ca^2+^-induced Ca^2+^ release.

### Components of the fast Ca^2+^ transient and their contribution to exocytosis

**Figure 3:**
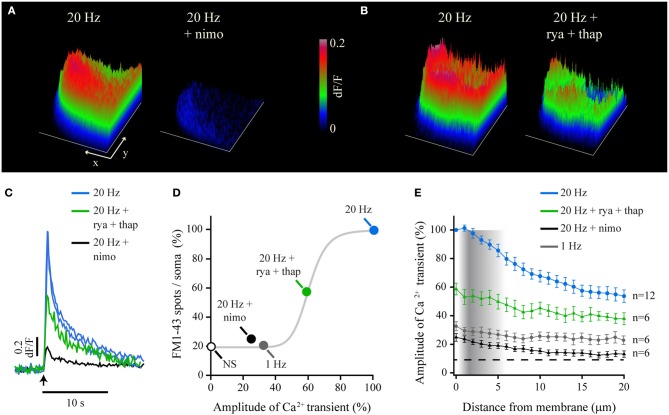
**Transmembrane and intracellular Ca^2+^ produce the fast Ca^2+^ transient and exocytosis. (A)** Surface plots of the peak Fluo-4 fluorescence upon 20-Hz stimulation before (left) and after (right) blockade of L-type Ca^2+^ channels with nimodipine (nimo). The x and y scale bars = 10 μm. The z-axis indicates the relative (dF/F) fluorescence levels as calibrated on the right bar (also applies for B). **(B)** Surface plots before (left) and after (right) blockade of Ca^2+^-induced Ca^2+^ release with a combination of ryanodine (rya) and thapsigargin (thap). **(C)** Superposition of the kinetics of representative Ca^2+^ transients recorded from the submembrane shell under the experimental conditions shown above. The blue traces are responses to control 20-Hz trains in the same neurons before addition of nimodipine (black trace) or the combination of ryanodine and thapsigargin (rya+thap respectively, green trace). The arrow indicates the onset of stimulation. **(D)** Percentage of exocytosis measured as the number of fluorescent FM1-43 spots/soma (Trueta et al., 2003, 2004), plotted as a function of the amplitude of the Ca^2+^ transient recorded under different experimental conditions and normalized to the amplitude of the transient produced by 20-Hz stimulation. The white dot indicates the number of spots/soma formed by non-stimulated neurons. The gray curve is a Hill sigmoidal function fitted to the data that empirically reproduces their trend. **(E)** Decay of the fast Ca^2+^ transient as it spreads toward the soma center. The density of the vertical gray bar is proportional to the density of vesicle clusters at rest, as measured from electron micrographs (data adapted from De-Miguel et al., 2012).

The pharmacological dissection of the components of the fast Ca^2+^ transient allowed us to understand its generation and spread, and provided us with tools to analyze how it evokes exocytosis. The fast Ca^2+^ transient had three components. The main component was a voltage-dependent L current, which was reduced by 75.1 ± 7.3% by the L-type Ca^2+^ channel blocker nimodipine (10 μM; Figures 3A,C). The same concentration of nimodipine produced a 75% reduction in the amount of somatic exocytosis, measured as the number of FM1-43 spots/soma in a different cell group (Trueta et al., 2003, see methods; *n* = 10; Figure 3D). This indicated that Ca^2+^ entry through L channels was the predominant source to trigger exocytosis. The 25% residual of the fast Ca^2+^ transient was insensitive to nimodipine at doses up to 80 μM, although was completely abolished when cadmium (200 μM) substituted Ca^2+^ in the external solution (not shown) to block transmembrane Ca^2+^ entry (Fernandez-De-Miguel et al., 1992). This experiment unveiled a second and yet unidentified transmembrane Ca^2+^ source. However, this remnant Ca^2+^ transient was unable to evoke exocytosis, since the 25% of fluorescent spots/soma counted in these conditions was in the range of the values counted after 1-Hz stimulation (20 ± 5%; *n* = 6), or after 20-Hz stimulation when Ca^2+^ had been replaced by magnesium in the external solution to prevent transmembrane Ca^2+^ entry (34 ± 8 %; *n* = 7; Figure 3D). Moreover, a 21% of spots/soma was produced in the absence of stimulation (*n* = 6).

As predicted before, the third Ca^2+^ source contributing to the fast transient was Ca^2+^-induced Ca^2+^ release. Incubation of six neurons with 100 μM ryanodine and 1.6 μM thapsigargin reduced the amplitude of the fast Ca^2+^ transient by 41 ± 5.1% (Figures 3B,C). Ca^2+^-induced Ca^2+^ release also contributed to exocytosis since pre-incubation of 11 neurons with 100 μm ryanodine (Figure 3D) in our previous experiments (Trueta et al., 2004) reduced the number of FM1-43 spots/soma by 42%. Figure 3D shows the percentage of fluorescent FM1-43 spots/soma plotted vs. the percentage peak amplitude of the fast Ca^2+^ transient obtained in each experimental condition. The 100% values were those obtained upon 20-Hz stimulation (see Methods). The gray sigmoidal function in Figure 3D shows the trend of the data for illustration purposes. This function was fitted to the data owing to its similarities to the plot describing how the amount of exocytosis depends on the stimulation frequency (Trueta and De-Miguel, in preparation). These data confirm on one hand that the amount of exocytosis is determined by the amplitude of the fast Ca^2+^ transient. On the other hand data show that different Ca^2+^ sources cooperate to determine the number of vesicle clusters arriving at the plasma membrane.

A further explanation to how the fast Ca^2+^ transient determines the amount of vesicle clusters undergoing exocytosis came from how far the fast Ca^2+^ transient penetrates the cytoplasm. This is shown by plotting the average amplitude of the fast Ca^2+^ transients in different experimental conditions over the radial intracellular distance from the plasma membrane (Figure 3E). The plot is superimposed to the intracellular density of vesicle clusters at rest, as estimated from electron micrographs (De-Miguel et al., 2012). It may be seen that the amplitude of the fast Ca^2+^ transient decayed within the distance range in which the vesicle clusters accumulate at rest. Therefore, peripheral clusters receive a higher Ca^2+^ concentration than internal clusters. Since vesicle clusters establish tight bounds with mitochondria (De-Miguel et al., 2012), an attractive possibility is that the amount of vesicle clusters transported toward the plasma membrane depends on amplitude of the fast Ca^2+^ transient through the activation of mitochondrial ATP synthesis. By sensing a larger Ca^2+^ concentration the more peripheral clusters would receive more ATP for their transport.

### The small and the large submembrane Ca^2+^ transients sustain exocytosis

**Figure 4:**
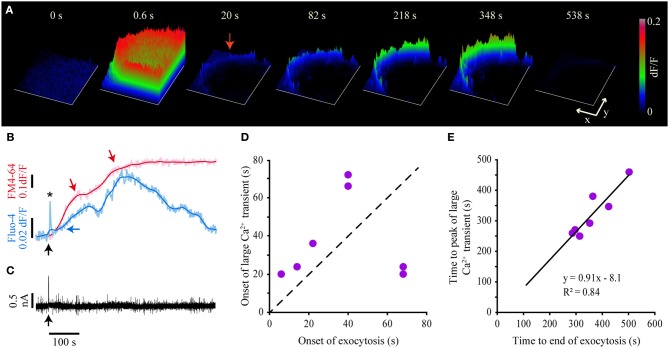
**Kinetics of the large Ca^2+^ transient and exocytosis. (A)** Sequence of surface plots of the Fluo-4 fluorescence in response to a 20-Hz train. The onset of the train was at *t* = 0. The frame at 0.6 s shows the peak of the fast Ca^2+^ transient; the frame at 20 s shows the small Ca^2+^ transient, and the subsequent frames show the development of the large Ca^2+^ transient. Note that the fluorescence increases were limited to the submembrane region of the soma. The x and y scale bars = 10 μm. The z-axis indicates the relative (dF/F) fluorescence levels as calibrated on the right bar. **(B)** Kinetics of simultaneously acquired FM4-64 (pink) and Fluo-4 (pale blue) confocal fluorescence following 20-Hz stimulation. The black arrow indicates the onset of the 20-Hz train. The kinetics of exocytosis had two sigmoidal components, each pointed by a red arrow. The smoothed kinetics (red and blue) are superimposed to each trace. The amplitude of the fast Ca^2+^ transient (asterisk) is truncated due to the 0.5-Hz imaging acquisition frequency. The Fluo-4 large transient started to rise after the onset of the FM4-64 increase (horizontal blue arrow) and developed two peaks, each of which matched with each plateau of the FM4-64 fluorescence kinetics. **(C)** The transmembrane current during the large Ca^2+^ transient remained constant after 20-Hz stimulation, thus eliminating the possibility that the large Ca^2+^ transient was produced by transmembrane Ca^2+^ entry. The arrow indicates the onset of the 20-Hz train. **(D)** The large Ca^2+^ transient appeared after the onset of the large-scale exocytosis, as seen by the points above the dashed identity bar. This relationship was inverted in two neurons with long exocytosis latencies (~70 s). **(E)** The peak of the large Ca^2+^ transient was reached 8 s before the end of exocytosis, as shown by the negative y-intercept of the linear relationship in the plot. The parameters of the fit are shown in the plot.

Since the large-scale exocytosis starts after the end of the fast Ca^2+^ transient, we investigated the possibility that exocytosis is sustained by the small and the large Ca^2+^ transients. As mentioned before and shown in Figure 4A, both Ca^2+^ transients were purely submembrane in the seven neurons studied (see also Movie 1). Figure 4B shows the kinetics of simultaneously acquired Fluo-4 and FM4-64 fluorescence. The two sigmoidal increases in the FM4-64 fluorescence correlate with the development of two Ca^2+^ transients. However, they were not produced by a transmembrane Ca^2+^ flow, since our voltage clamp records during exocytosis lacked any transmembrane currents (Figure 4C).

The small Ca^2+^ transient could be detected 6.6–24.0 s after the onset of the train (Figure 2D, arrowhead). By contrast, the large Ca^2+^ transient (Figures 4A,B) was only detected 14–35 s after the onset of the large-scale exocytosis (determined as the threshold for the FM4-64 fluorescence increase) in five out of seven neurons; that is 21–72 s after the onset of the stimulation train (Figure 4D). In two other neurons in which the onset of exocytosis had a longer latency (>68 s), the large Ca^2+^ transient anticipated exocytosis. However, in every neuron tested the large Ca^2+^ transient grew during the dynamic range of exocytosis to reach peak fluorescence levels 638–992% above the baseline. The decay of the transient started 8 s before the end of exocytosis, when its rate had started to decline (Figures 4B,E). This value was calculated from the y-intercept of the line that fitted the time to peak of the large Ca^2+^ transient vs. the duration of exocytosis (Figure 4E). The small and the large submembrane Ca^2+^ transients were smaller and had a slower time course in submembrane segments that failed to develop FM4-64 fluorescent spots, thus suggesting that the intracellular Ca^2+^ had diffused from active exocytosis sites. These observations suggested that both submembrane Ca^2+^ transients received an input from exocytosis.

### 5-HT release and phospholipase C activation produced the submembrane Ca^2+^ transients and exocytosis

**Figure 5:**
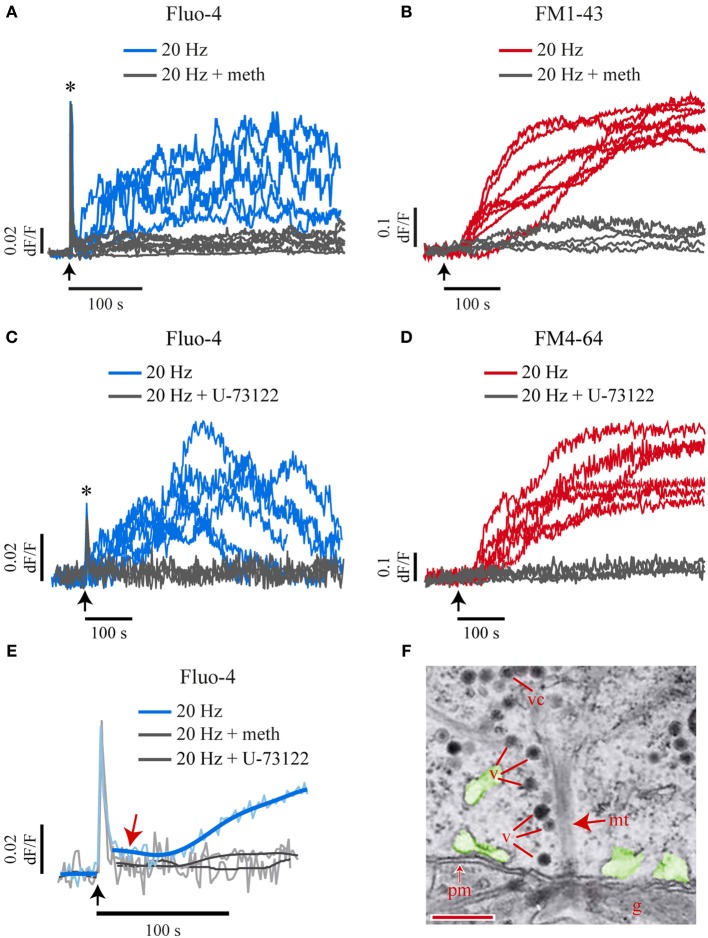
**Extracellular 5-HT and PLC activation produce the small and the large Ca^2+^ transients and exocytosis. (A)** Kinetics of Fluo-4 fluorescence in response to a 20-Hz train in the absence (blue traces) or presence (gray traces) of methysergide (140 μM) to block the activation of 5-HT receptors. This treatment abolished the large Ca^2+^ transient (*n* = 6) without affecting the fast Ca^2+^ transient (see asterisk). The arrows indicate the onset of stimulation. **(B)** Methysergide abolished the FM1-43 fluorescence increases in the six neurons tested (gray traces). The kinetics shown in red were obtained from other neurons in the absence of methysergide. **(C,D)** Blockade of PLC with U-73122 (10 μm) abolished the submembrane Fluo-4 **(C)** and FM4-64 **(D)** fluorescence increases in response to 20-Hz stimulation (gray traces) without affecting the fast Ca^2+^ transient (which appears truncated due to the 0.5-Hz imaging sampling rate; asterisk). Exocytosis and Ca^2+^ increases were measured simultaneously under confocal conditions. **(E)** Blockade of 5-HT receptors or PLC (gray traces) also abolished the small Ca^2+^ transient (blue trace, red arrow). The pale blue traces are an average of the Ca^2+^ kinetics obtained from seven neurons. Smoothed traces are superimposed in darker blue. **(F)** Electronmicrograph showing dense core vesicles (v) aligned to the microtubule bundles (mt) arriving at the plasma membrane (pm) of a soma that was fixed after 1-Hz stimulation. Exocytosis from these vesicles may release the 5-HT that produces the small Ca^2+^ transient. The endoplasmic reticulum, possible intracellular Ca^2+^ source is pseudocolored in green. The glia is marked as “g.” Scale bar = 200 nm.

To explore if the small and the large submembrane Ca^2+^ transients were produced by 5-HT released upon exocytosis, neurons were stimulated with a 20-Hz train in the presence of methysergide (140 μM), an unspecific blocker of metabotropic 5-HT inverterbrate receptors. Methysergide abolished both submembrane Ca^2+^ transients without affecting the fast Ca^2+^ transient in the six neurons stimulated at 20 Hz (Figure 5A). In six other stimulated neurons methysergide abolished exocytosis (Figure 5B). These results indicated that 5-HT exocytosis activates autoreceptors, which then produce the submembrane Ca^2+^ transients that evoke more exocytosis. Consistently, iontophoretic applications of 5-HT to the soma surface produced a Fluo-4 fluorescence increase in the submembrane area adjacent to the iontophoretic pipette (Supplementary Figure 2). By contrast, a bath perfusion with 1 μM 5-HT, a concentration that in our hands produces several physiological effects without evoking action potentials in Retzius neurons (Sarkar et al., 2014), failed to evoke any exocytosis (n=6 neurons). These results suggest that the 5-HT-evoked submembrane Ca^2+^ transients sustain the large-scale somatic exocytosis, but the fast Ca^2+^ transient is required as a trigger.

We next investigated the mechanism by which the released 5-HT activates the small and the large submembrane Ca^2+^ transients. Although other experiments made in these neurons have shown brief transmembrane currents in response to 5-HT pulses (Lessmann and Dietzel, 1991; Beck et al., 2002), the lack of transmembrane currents in our records during the large scale 5-HT exocytosis (Figure 4C) suggested that both submembrane Ca^2+^ transients were produced by intracellular Ca^2+^ release. Extrasynaptic 5-HT_2_ receptors in Retzius neurons are coupled to phospholipase C (PLC) (Drapeau et al., 1989; Sanchez-Armass et al., 1991), which produces inositol 1,4,5-triphosphate (IP_3_) and intracellular Ca^2+^ release (Pozzan et al., 1994; Pandey et al., 1995; Barnes and Sharp, 1999). This pathway was then explored as the possible origin of the submembrane Ca^2+^ transients by blocking the activation of PLC with U-73122 (10 μM; Jin et al., 1994). In simultaneous confocal imaging of FM4-64 and Fluo-4 fluorescence (*n* = 7 neurons) this manipulation abolished both submembrane Ca^2+^ transients and exocytosis upon 20-Hz stimulation. However, the fast Ca^2+^ transient remained intact (Figures 5C–E).

These results provided two lines of information. On one hand, they confirmed that 5-HT binding to autoreceptors activates PLC and intracellular Ca^2+^ release. On the other hand, they gave evidence that the small Ca^2+^ transient is also evoked by the release of small amounts of 5-HT which in our experimental conditions were undetectable as FM dye fluorescence increases. This hypothesis was supported by the presence of vesicles aligned to microtubules that linked vesicle clusters with the plasma membrane. These observations came from 16 electron micrographs obtained from five neurons fixed at rest or after 1-Hz stimulation (Figure 5F, see also Supplementary Figure 1). Therefore, we suppose that an early exocytosis from these vesicles in response to the fast calcium transient produces the small Ca^2+^ transient while the vesicle clusters arrive at the plasma membrane.

### Somatic exocytosis is sustained by a Ca^2+^- and 5-HT-dependent positive feedback loop

**Figure 6:**
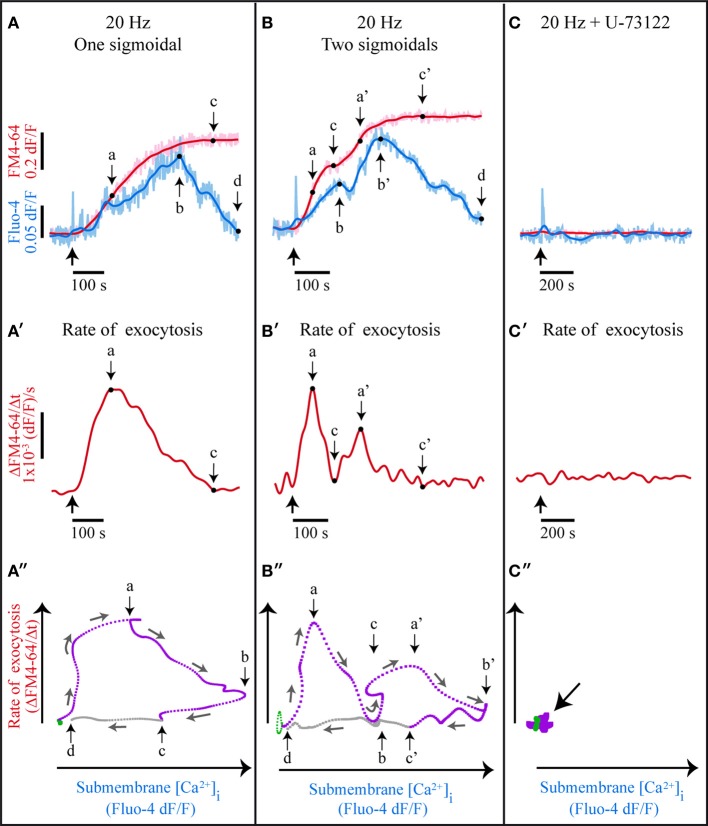
**A bistable system with a positive feedback loop sustains somatic exocytosis. (A)** Kinetics of simultaneously acquired Fluo-4 (pale blue) and FM4-64 (pink) confocal fluorescence in response to 20-Hz stimulation. The smoothed kinetics are superimposed (dark blue and red traces, respectively). The thick black arrow indicates the stimulation onset; “a” indicates the maximal rate of exocytosis, as estimated from the differential of the kinetics shown in **(A′)**; “b” is the peak of the large Ca^2+^ transient; “c” is the end of exocytosis, when the differential value of the FM4-64 dye fluorescence is zero (see **A′**); “d” indicates the end of the large Ca^2+^ transient. **(B)** Exocytosis and Ca^2+^ kinetics displaying two sigmoidal increases upon subsequent exocytosis from two vesicle clusters. The large Ca^2+^ transient also displays two peaks. “a**'**–c**'**” are as in **(A)**, although applied to the second sigmoidal kinetics. **(C)** In the presence of U-73122 only the fast Fluo-4 transient persisted. **(A′–C′)** Rates of exocytosis obtained from the differential of FM4-64 fluorescence kinetics of the data above. **(B′)** The rate of exocytosis displays two peaks, each indicating exocytosis from one vesicle cluster. In **(C′)** the rate of exocytosis was zero. **(A″–C″)** Rate of exocytosis plotted vs. the peak Ca^2+^ concentration. **(A″)** At rest the cells are in an off-state (green) and upon electrical stimulation they transit to a dynamic on-state (purple). The small arrows indicate the time course of the sequence. The feedback loop is active as long as 5-HT is released (from the origin of the purple line to point “c”). When Ca^2+^ returns to its basal levels (point “d”) the system returns to the off-state. **(B″)** The rate of exocytosis as a function of the Ca^2+^ concentration also displays two subsequent cycles. **(C″)** blockade of PLC stops the transition from the off to the on-state.

A formal documentation that somatic 5-HT exocytosis is maintained by a positive feedback loop came from plotting the rate of exocytosis (the differential of the FM4-64 fluorescence over time Figures 6A′–C′) vs. the intracellular Ca^2+^ concentration (the Fluo-4 fluorescence over time). The cyclic shape of the plots shown in Figure 6 is characteristic of a bistable system in a dynamic state, sustained by a positive feedback loop (Gardner et al., 2000; Becskei, 2001; Qu and Vondriska, 2009). A 20 Hz train switches the system from a resting “off-state” (green traces in Figures 6A″,B″) to a dynamic exocytosis “on-state” (purple traces in Figure 6A″) by triggering transmembrane Ca^2+^ entry and exocytosis. The feedback loop operates during the on-state, when the released 5-HT produces the submembrane Ca^2+^ transients and these transients evoke more exocytosis. The intracellular Ca^2+^ concentration declines when exocytosis fades out, and the feedback loop ends upon exocytosis from the last vesicles in the cluster (letters “c” and “c'” Figure 6). The system goes back to the off-state (letters “d” in Figure 6) when the Ca^2+^ concentration returns to its basal level (gray interval between “c” and “d” in Figures 6A″,B″).

Those FM4-64 fluorescent spots that displayed a second sigmoidal kinetics also described a second submembrane Ca^2+^ peak (Figure 6B) and a second dynamic cycle in the plots (Figure 6B″). The new cycle expressed a new activation of the feedback loop, thus being consistent with the evidence on the fusion of vesicles arriving in a second cluster (De-Miguel et al., 2012). Note in Figure 6B″ that the second cycle started in the absence of new electrical stimulation, when the submembrane Ca^2+^ concentration was still high and the system remained in the on-state. As expected, when the 5-HT receptors or PLC were blocked (Figures 6C–C″) the system could not transit from the off- to the on-state.

## Discussion

**Figure 7:**
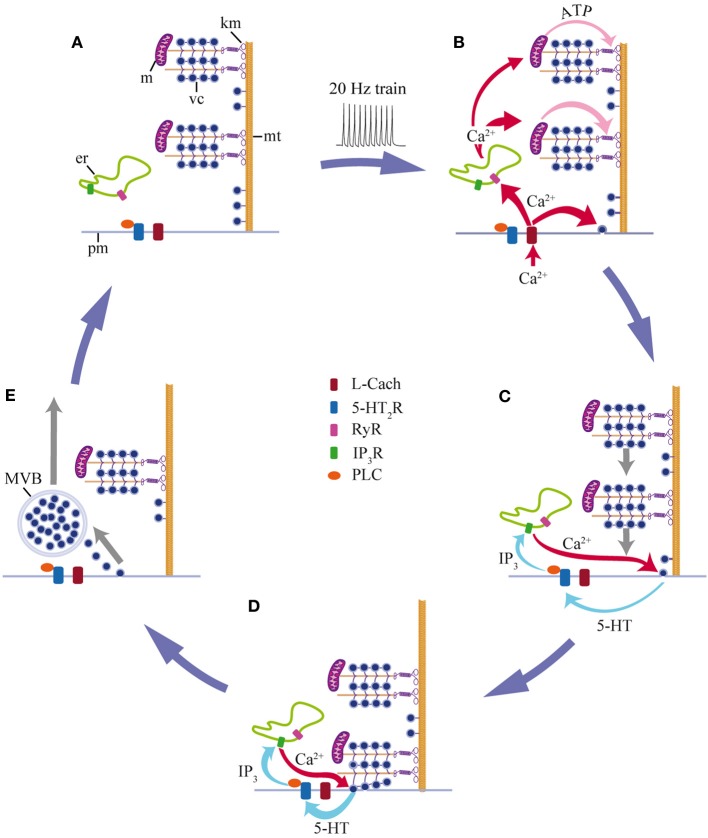
**Schematic representation of the mechanism for somatic 5-HT exocytosis by Retzius neurons. (A)** At rest, vesicle clusters (vc) and mitochondria (m) are distant from the plasma membrane. Both are attached to microtubules (mt) that arrive at the plasma membrane (pm). Endoplasmic reticulum (er) rests between the plasma membrane and the vesicle clusters. **(B)** A train of impulses evokes transmembrane Ca^2+^ entry through L-type channels (L Cach). Ca^2+^ triggers exocytosis from vesicles that are close to the plasma membrane and in parallel, Ca^2+^ entry activates ryanodine receptors (RyR) and Ca^2+^-induced Ca^2+^ release, presumably from endoplasmic reticulum. The fast Ca^2+^ transient reaches the mitochondria (m), which responds by producing ATP. Kinesin motors (km) become activated. **(C)** Vesicle clusters are transported toward the plasma membrane. The peripheral vesicle clusters and mitochondria receive more Ca^2+^ and ATP than the central clusters. Therefore, they are transported more efficiently toward the plasma membrane. As vesicles arrive at the plasma membrane and fuse, the released 5-HT activates 5HT_2_ receptors (5HT_2_R) and triggers an intracellular cascade. PLC activation induces IP_3_ production; IP_3_ acts on its receptors (IP_3_R) and activates intracellular Ca^2+^ release from the submembrane endoplasmic reticulum (ER). This Ca^2+^ evokes more exocytosis, thus closing the local feedback loop. **(D)** Arrival of the vesicle clusters at the plasma membrane gives place to the large-scale exocytosis. **(E)** Exocytosis and the feedback loop end when the last vesicles in the cluster fuse. Endocytosis produces multivesicular bodies (MVB) that are transported back to perinuclear regions of the soma (adapted from Trueta et al., 2012). The Ca^2+^ levels return to rest and the system goes back to its off-state **(A)**. Data from this study are complemented with data from De-Miguel et al., (2012); Trueta et al., (2012).

We have shown that a brief stimulation with 10 impulses at 20 Hz produces a fast Ca^2+^ transient that triggers exocytosis and determines its magnitude. The delayed large-scale exocytosis is then maintained through a positive feedback loop in which the released 5-HT activates 5-HT_2_ autoreceptors coupled to PLC. This produces a submembrane Ca^2+^ elevation that triggers more exocytosis. The feedback loop ends upon fusion of the last vesicles in the clusters that arrived at the plasma membrane in response to stimulation. The detailed steps of somatic 5-HT exocytosis shown in this study and complemented with data from our previous work (Trueta et al., 2003, 2004; De-Miguel et al., 2012; Trueta et al., 2012) are summarized in Figure 7.

### Multiplicity of Ca^2+^ effects on somatic exocytosis

Summation of Ca^2+^ entering across the plasma membrane may explain how the stimulation frequency determines the amount of intracellular Ca^2+^ and exocytosis. The slow inactivation of L-type channels supports a continuous Ca^2+^ entry throughout the stimulation train (Nowycky et al., 1985; Fox et al., 1987; Tsien et al., 1988). Since the amplitude of the L-type Ca^2+^ currents in Retzius neurons remains similar along subsequent impulses (Ross et al., 1987; Stewart et al., 1989), a temporal summation of the Ca^2+^ entering the soma upon the successive 20-Hz impulses may produce a rapid and large increase in the Ca^2+^ concentration as the stimulation frequency is increased. This summation along with the wide sensitivity to Ca^2+^ of the ryanodine receptors, which increase their opening probability sigmoidally at [Ca^2+^] between 0.1 and 100 μm (Bezprozvanny et al., 1991), explains why increasing the stimulation frequency activates Ca^2+^-induced Ca^2+^ release. A similar combination of transmembrane Ca^2+^ entry and Ca^2+^-induced Ca^2+^ release produces dendrosomatic exocytosis in hypothalamic neurons releasing oxytocin (Ludwig et al., 2002; Tobin et al., 2011, 2012) and in substantia nigra neurons releasing dopamine (Patel et al., 2009), thus suggesting that a similar mechanism operates in those neurons.

Somatic exocytosis in Retzius neurons depends on a microtubule-based vesicle transport (De-Miguel et al., 2012). Indirect evidence suggests a that a similar phenomenon occurs in other neuron types (Jaffe et al., 1998; Puopolo et al., 2001; Huang et al., 2007; Zhang et al., 2007; Hirasawa et al., 2009; Sarkar et al., 2012). This transport is mediated by molecular motors and therefore somatic exocytosis depends indirectly on ATP synthesis (Bi et al., 1997; Visscher et al., 1999). Ca^2+^-induced Ca^2+^ release may then determine the amount of vesicle clusters transported and fused by increasing the intracellular Ca^2+^ concentration to levels that activate mitochondrial ATP synthesis (Hansford, 1994; Balaban, 2002; Gunter et al., 2004; Satrústegui et al., 2007). In chromaffin cells depolarization and Ca^2+^ entry also produce the active transport of dense core vesicles before their fusion (Oheim and Stühmer, 2000; Becherer et al., 2003). Also in chromaffin cells dense core vesicles are important sources for Ca^2+^-induced Ca^2+^ release (Mitchell et al., 2001; Moreno et al., 2005; Yoo, 2010). However, our results do not support a similar vesicular Ca^2+^ release in Retzius neurons, since the amplitude of the fast Ca^2+^ transient in the regions heavily populated with vesicle clusters continues to decay monotonically on its way toward the soma center. As a consequence of the Ca^2+^ concentration decay as the transient spreads, the peripheral assemblies of mitochondria and vesicle clusters receive more Ca^2+^ than their equivalents resting more internally. Therefore, the molecular motors associated to these external vesicle clusters would receive more newly-synthesized ATP and therefore, they would be more efficiently transported towards the plasma membrane than those clusters resting internally. In this way the number of vesicle clusters transported and fused may be proportional to the amplitude of the fast Ca^2+^ transient.

### The beginning and end of somatic exocytosis

Somatic exocytosis starts when the fast Ca^2+^ transient triggers exocytosis from small numbers of vesicles resting near the plasma membrane. This exocytosis triggers the feedback loop. A delayed arrival of vesicle clusters at the “activated” release regions produces the large-scale exocytosis by increasing the magnitude of the feedback loop. Therefore, the small and the large Ca^2+^ transients are expressions of the same phenomenon, namely the 5-HT-and Ca^2+^-dependent feedback loop that sustains exocytosis. The strict submembrane localization of these Ca^2+^ transients allows a continuous exocytosis without triggering any further vesicle transport from internal regions. The localization of these transients correlates with the presence of large bags of endoplasmic reticulum near the plasma membrane in electron micrographs. The function of such highly regulated and localized Ca^2+^ increase prevents a depletion of the somatic 5-HT pool by preventing further activation of vesicle transport from internal somatic regions. However, we have shown that in some cases, a second round of exocytosis occurred in the absence of additional electrical stimulation, provided that the large submembrane Ca^2+^ transient had not ended. This suggests that a single 20 Hz train also induces the transport of more distal clusters by the same microtubule rails. In these vesicle clusters approach but do not always reach the plasma membrane, they would constitute the future releasable pool in response to a new train of impulses.

It is interesting that each vesicle cluster functions as an “exocytosis unit” with all its vesicles releasing their content once exocytosis it is triggered, since it allows the all-or-none release of large amounts of 5-HT over long periods. Moreover, exocytosis from the last vesicles in the cluster confers an end to the large-scale exocytosis and to the large Ca^2+^ transient. A similar phenomenon may occur in the soma of raphe and substancia nigra neurons, in which depolarization induces exocytosis from structures with diameters similar to those of the vesicle clusters of Retzius neurons and also with a similar time course (Sarkar et al., 2012, 2014).

### Integration of a feedback system to regulate somatic exocytosis

Positive feedback loops constitute dynamic bistable systems which allow a brief stimulus (such as the 20-Hz trains used here) to produce a robust response that may persist over long periods in the absence of any further stimulation (Qu and Vondriska, 2009). At the cellular level positive feedback loops contribute to the regulation of intracellular messenger cascades (Gardner et al., 2000; Xiong and Ferrell, 2003; Qu and Vondriska, 2009), the release of hormones (Sabatier et al., 2003; Wintermantel et al., 2006) and cell-to-cell communication in the retina (Jackman et al., 2011). The mechanism presented here is strikingly sophisticated, since it involves multiple steps, the contribution of different intracellular organelles, three Ca^2+^ sources and events occurring inside and outside the soma. This may be a mechanism of general significance in biology, since in neuroepithelial body cells of rodents; hypoxia produces a 5-HT- and Ca^2+^-dependent increase in 5-HT release (Fu et al., 2002). Moreover, in dendrites of hypothalamic neurons the activation of dendritic oxytocin autoreceptors elevates the intracellular Ca^2+^ concentration and triggers exocytosis from dense core vesicles (Lambert et al., 1994; Neumann et al., 1996; Ludwig and Leng, 2006).

### Physiological importance of somatic exocytosis for serotonergic systems

The 5-HT modulation of circuits and behaviors along phylogeny requires large amounts of transmitter molecules acting at different levels of the nervous system (Saller and Stricker, 1976; Willard, 1981; Lent, 1985; Raleigh et al., 1991; Gillette et al., 1993; Barnes and Jacklet, 1997; Hull et al., 1999; Kravitz, 2000; Prosser, 2003; Alekseyenko et al., 2010; Tomioka et al., 2012). Somatic exocytosis seems particularly well fitted for this function. In the leech, electrical stimulation of Retzius neurons with high-frequency trains of impulses increases the levels of extracellular 5-HT in the ganglion and in the blood (Willard, 1981). This stimulation also produces a non-synaptic 5-HT activation of the swimming circuit (Nusbaum and Kristan, 1986), thus suggesting that it occurs in response to somatic and maybe axonal 5-HT release. In mammals, serotonergic neurons innervate most of the central nervous system (Jacobs and Azmitia, 1992) and 5-HT is released through somatic and dendritic exocytosis (De Kock et al., 2006; Kaushalya et al., 2008; Colgan et al., 2009, 2012; Sarkar et al., 2012). This release in addition to the small numbers of presynaptic terminals formed by serotonergic neurons (Mosko et al., 1977; Héry and Ternaux, 1981; Chazal and Ralston, 1987; Marlier et al., 1991; Ridet et al., 1993; Moukhles et al., 1997) suggests that somatic and in general extrasynaptic exocytosis is a major player of 5-HT communication (Agnati et al., 1986; De-Miguel and Fuxe, 2012; Fuxe et al., 2012; Trueta and De-Miguel, 2012). It was already mentioned that the soma of raphe neurons releases 5-HT for long periods from structures that may be similar to the clusters shown here (Kaushalya et al., 2008; Sarkar et al., 2012). In addition, the dendrites and axons of these neurons contain clear and dense core vesicles in arrangements suitable to produce extrasynaptic exocytosis (Kapadia et al., 1985; Liposits et al., 1985; Chazal and Ralston, 1987; Ridet et al., 1993; Van Bockstaele and Pickel, 1993; Bunin and Wightman, 1999; Descarries and Mechawar, 2000). Moreover, in these neurons the activation of L-type channels evokes dendritic exocytosis (Colgan et al., 2012). These evidences suggest that the mechanism shown here may also operate at smaller volume- and time-scales in dendrites and axons of mammalian serotonergic neurons. Such idea is consistent with the 5 s latency of 5-HT extracellular increases upon dendro-somatic stimulation in rat brain slices (Bunin and Wightman, 1998).

It is interesting to note that 5-HT autoreceptors are typically inhibitory and belong to the 5-HT_1_type (for review see Barnes and Sharp, 1999). However, the autoreceptors involved in the positive feedback system shown here are of the 5-HT_2_-type, according to their pharmacological profile and intracellular metabolic cascade (Drapeau et al., 1989; Sanchez-Armass et al., 1991; Barnes and Sharp, 1999). The incorporation of this type of receptor may be an adaptation of somatic exocytosis, since presynaptic terminals formed by Retzius neurons contain the characteristic 5-HT_1_ type receptors that inhibit electrical activity and transmitter release (Cercós et al., 2009), similar to their function in mammals (Chaput et al., 1986; Trulson and Frederickson, 1987; O'Connor and Kruk, 1991; Fornal et al., 1994).

Two additional aspects are worth discussing. One is that the decay time course of the large submembrane Ca^2+^ transient may be due, at least in part, to the removal of 5-HT from the extracellular space. The other is that 5-HT release in the intact nervous system occurs onto the extracellular space which is tightly surrounded by a giant glial cell (Coggeshall and Fawcett, 1964; Kuffler and Nicholls, 1966; see also Supplementary Figure 1). Since this glial cell has 5-HT an uptake system (Bruns et al., 1993), it may contribute to the 5-HT removal from the extracellular space. Therefore, these transporters may modulate the duration of exocytosis. On the other hand, the virtual isolation of the soma of the Retzius neuron by the glial sheets suggests that the 5-HT uptake participates in the distribution of 5-HT to other sites of the nervous system.

### General significance

Somatic exocytosis is a widespread mechanism for paracrine communication in the nervous system (De-Miguel and Fuxe, 2012; Fuxe et al., 2012; Trueta and De-Miguel, 2012), and evidence suggests that transmitters released in this way modulate the activity of neurons, glia and blood vessels (Fuxe et al., 1988; Descarries and Mechawar, 2000; Jansson et al., 2001; Del Arco et al., 2003; Fuxe et al., 2010; Thyssen et al., 2010; De-Miguel and Fuxe, 2012). The similar duration of somatic exocytosis in several neuron types (De-Miguel and Fuxe, 2012; Trueta and De-Miguel, 2012) suggest that somatic exocytosis it is ruled by a similar mechanism. Therefore, the findings presented here may be of general interest to explain the cellular basis of paracrine communication in the nervous system.

## Author contributions

The author(s) have made the following declarations about their contributions: Carolina Leon-Pinzon and Francisco F. De-Miguel conceived and designed the experiments. Carolina Leon-Pinzon, Montserrat G. Cercós, Paula Noguez and FFM performed the experiments. Carolina Leon-Pinzon, Montserrat G. Cercós, Citlali Trueta and Francisco F. De-Miguel analyzed the data. Francisco F. De-Miguel and Citlali Trueta contributed reagents/materials/analysis tools. Francisco F. De-Miguel and Carolina Leon-Pinzon wrote the paper.

### Conflict of interest statement

The authors declare that the research was conducted in the absence of any commercial or financial relationships that could be construed as a potential conflict of interest.
