# Supplementary material for: Exocytosis of serotonin from the neuronal soma is sustained by a serotonin and calcium-dependent feedback loop
Source: Front Cell Neurosci. 2014 Jun 27;8:169. doi: 10.3389/fncel.2014.00169 (PMC4072984; doi:10.3389/fncel.2014.00169)
Supplement: Supplementary file 3 [file DataSheet1.PDF]

Supplementary Material

Exocytosis of serotonin from the neuronal soma is sustained by a serotonin and calcium-dependent feedback loop

Carolina Leon-Pinzon[1], Montserrat G. Cercós[2], Paula Noguez[1], Citlali Trueta[2] and Francisco F. De-Miguel[1*]

[1] Instituto de Fisiología Celular-Neurociencias, Universidad Nacional Autónoma de México, D.F., México.
[2] Instituto Nacional de Psiquiatría Ramón de la Fuente Muñiz, Departamento de Neurofisiología México D.F., México.

[*] **Correspondence:** Dr. Francisco F. De-Miguel, Instituto de Fisiología Celular-Neurociencias, Universidad Nacional Autónoma de México, Circuito Exterior, Ciudad Universitaria, 04510, Apartado Postal 14-740. C.P. 04510, México D.F., México. ffernand@ifc.unam.mx

1. Supplementary Figure 1

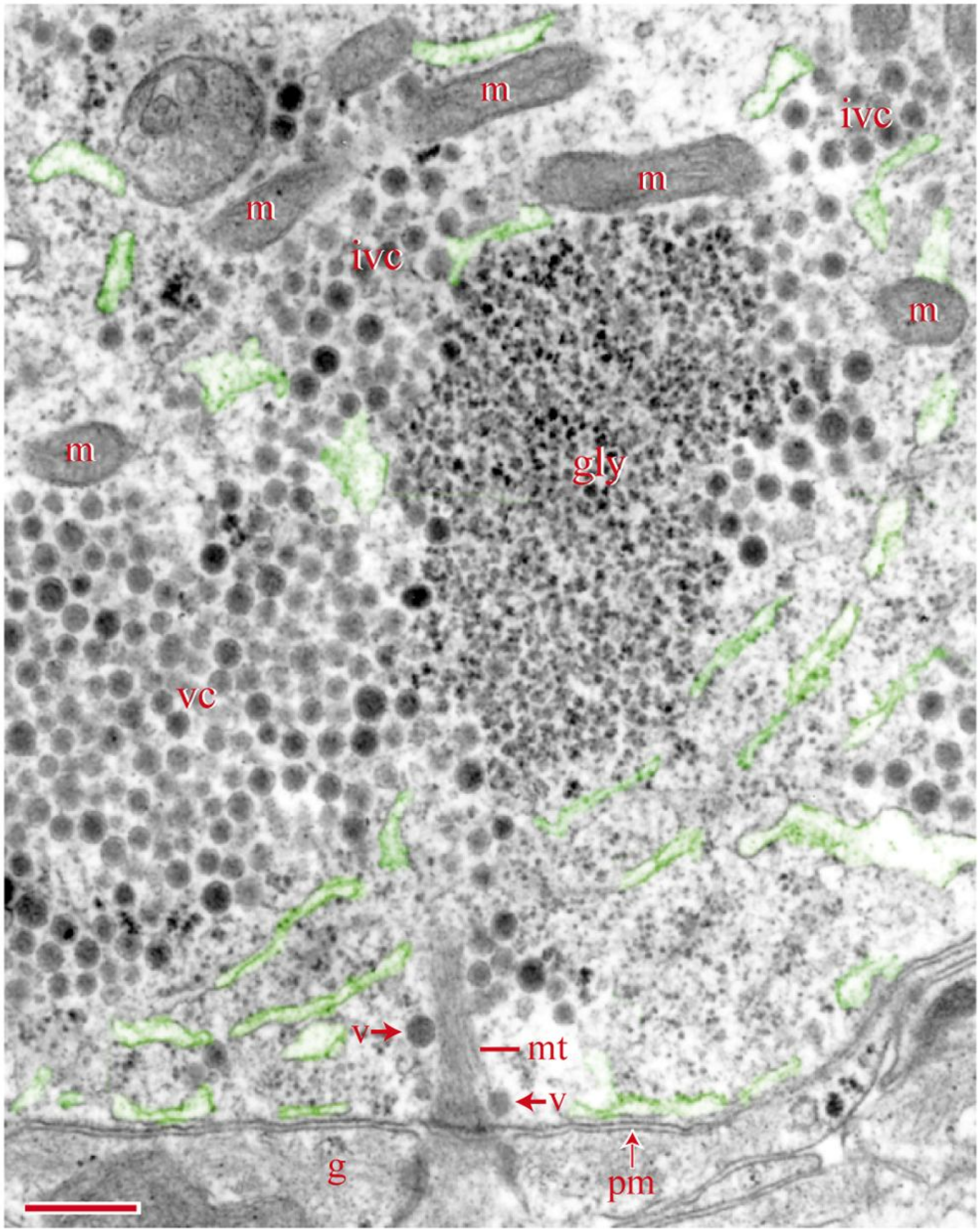

Supplementary Figure 1. Ultrastructure of a somatic release site. Electron micrograph of the equatorial region of a Retzius neuron fixed 10 minutes after being stimulated with 10 trains at 1 Hz. Vesicle clusters (vc) remained at a distance from the plasma membrane (pm). The upper part of the image shows a more internal vesicle cluster (ivc). Exocytosis from clusters like these may produce the second FM dye fluorescence increase. Microtubule bundles (mt) with individual associated vesicles (v) connect the vesicle clusters with the plasma membrane. The intracellular space between the vesicle clusters and the plasma membrane contains endoplasmic reticulum (pseudocolored green), which may be the main intracellular Ca^{2+}-releasing structure contributing to somatic exocytosis. Mitochondria (m) and endoplasmic reticulum can be seen on top of the vesicle clusters. A glycogen deposit (gly) appeared by the center of the image. The soma is surrounded by

layers of a glial cell (g) onto which exocytosis occurs. Scale= 250 nm.

2. Supplementary Movie 1

Movie 1. Intracellular Ca^{2+} transients evoked by a 20-Hz train. Movie made from a sequence of Fluo-4 fluorescence surface plots from an equatorial image of the soma (see Figure 2) upon 20-Hz stimulation. Subsequent images were taken every 100 ms. The x and y axes are the coordinates of the image. The z axis is the relative change in fluorescence intensity. The time is in yellow. Stimulation consisted of 10 impulses at 20 Hz (lasting 500 ms) applied at 0 s. Note that the transient fluorescence increase reached the center of the soma. The decay of the fast transient is followed by a submembrane intermediate transient.

3. Supplementary Movie 2

Movie 2. Intracellular Ca^{2+} transients evoked by a 1-Hz train. Movie made from a sequence of Fluo-4 fluorescence surface plots from the same neuron shown in Movie S1. The acquisition conditions are the same as for Movie S1. Note that in this case the stimulation train lasted 10 s and each impulse produced a submembrane fluorescent transient.

4. Supplementary Figure 2

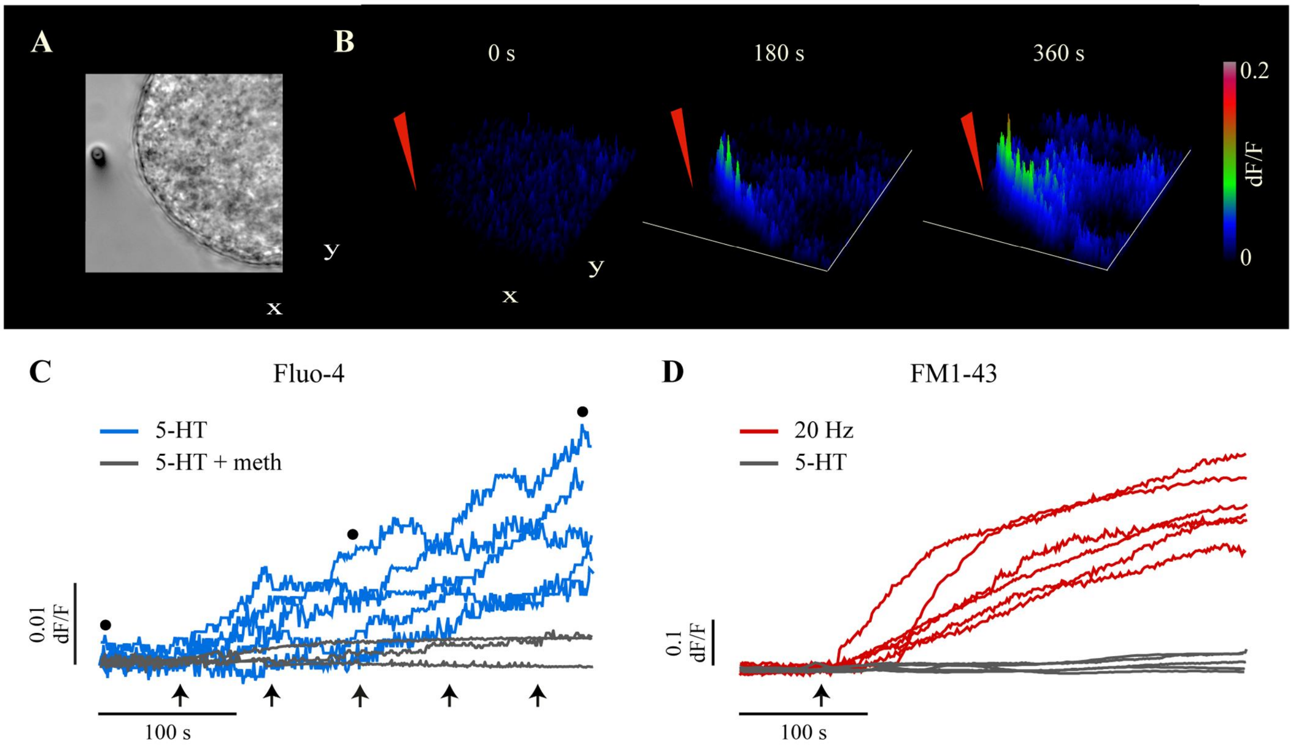

Supplementary Figure 2. External application of 5-HT produces slow submembrane Ca^{2+} transients without evoking large-scale somatic exocytosis. **(A)** Bright field equatorial image of the soma. The spot indicates the site from which Ca^{2+} increases were measured. The black spot outside the soma (pointed by the red arrowhead) is the tip of the 5-HT-containing electrode. Scale bar = 10 µm also applies to B. **(B)** Sequence of surface plots of Fluo-4 fluorescence increases evoked by 20-Hz trains of 5-HT iontophoretic application lasting 0.5 s. Individual pulses had a 10 nA amplitude and a 20 ms duration. One train was applied every 2 minutes. The position of the 5-HT electrode is indicated by the red symbol. **(C)** Increases in the submembrane Fluo-4 fluorescence induced by iontophoretic 5-HT application (black arrows) in six somata. Methysergide (140 µm) blocked the 5-HT-induced fluorescence increases (grey traces). The dots indicate the moment at which images in B were taken. Arrows indicate the time of 5-HT application. **(D)** Bath application of 1 µM exogenous 5-HT failed to evoke exocytosis (grey traces). Traces obtained upon 20-Hz stimulation (red) in six other neurons from the same batch are presented for comparison. The arrow indicates the onset of electrical stimulation.
